# Trypanocide Treatment of Women Infected with *Trypanosoma cruzi* and Its Effect on Preventing Congenital Chagas

**DOI:** 10.1371/journal.pntd.0003312

**Published:** 2014-11-20

**Authors:** Diana L. Fabbro, Emmaria Danesi, Veronica Olivera, Maria Olenka Codebó, Susana Denner, Cecilia Heredia, Mirtha Streiger, Sergio Sosa-Estani

**Affiliations:** 1 Centro de Investigaciones sobre Endemias Nacionales (CIEN) - Facultad de Bioquímica y Ciencias Biológicas- Universidad Nacional del Litoral, Santa Fe, Argentina; 2 Centro Nacional de Diagnóstico e Investigaciones Endemo-epidemicas, Administración Nacional de Laboratorios e Institutos de Salud (ANLIS), Buenos Aires, Argentina; 3 Instituto Nacional de Parasitología (INP), “Dr Mario Fatala Chaben”, Administración Nacional de Laboratorios e Institutos de Salud (ANLIS) Malbrán, Buenos Aires, Argentina; Institute of Tropical Medicine, Belgium

## Abstract

With the control of the vectorial and transfusional routes of infection with *Trypanosoma cruzi*, congenital transmission has become an important source of new cases. This study evaluated the efficacy of trypanocidal therapy to prevent congenital Chagas disease and compared the clinical and serological evolution between treated and untreated infected mothers. We conducted a multicenter, observational study on a cohort of mothers infected with *T. cruzi*, with and without trypanocidal treatment before pregnancy. Their children were studied to detect congenital infection. Among 354 “chronically infected mother-biological child” pairs, 132 were treated women and 222 were untreated women. Among the children born to untreated women, we detected 34 infected with *T. cruzi* (15.3%), whose only antecedent was maternal infection. Among the 132 children of previously treated women, no infection with *T. cruzi* was found (0.0%) (p<0.05). Among 117 mothers with clinical and serological follow up, 71 had been treated and 46 were untreated. The women were grouped into three groups. Group A: 25 treated before 15 years of age; Group B: 46 treated at 15 or more years of age; Group C: untreated, average age of 29.2±6.2 years at study entry. Follow-up for Groups A, B and C was 16.3±5.8, 17.5±9.2 and 18.6±8.6 years respectively. Negative seroconversion: Group A, 64.0% (16/25); Group B, 32.6% (15/46); Group C, no seronegativity was observed. Clinical electrocardiographic alterations compatible with chagasic cardiomyopathy: Group A 0.0% (0/25); B 2.2% (1/46) and C 15.2% (7/46). The trypanocidal treatment of women with chronic Chagas infection was effective in preventing the congenital transmission of *Trypanosoma cruzi* to their children; it had also a protective effect on the women's clinical evolution and deparasitation could be demonstrated in many treated women after over 10 years of follow up.

## Author Summary

Congenital infection with *Trypanosoma cruzi* is the most common mode of transmission in countries where the vectorial and transfusional routes have been controlled. We evaluated the efficacy of trypanocidal therapy in preventing congenital Chagas disease and compared the clinical and serological changes between treated and untreated mothers. From medical records of infected women with and without trypanocidal treatment, we studied their children to detect congenital infection. Among 222 children of untreated mothers, we detected 34 cases of congenital transmission. The 132 children of previously treated women were born without infection. In 117 mothers followed for more than eight years, we observed negative serology for Chagas in 43.7% of the treated women and none in untreated mothers. Regarding their clinical course, there was also a positive difference for treated women (2.2%), compared with untreated ones (15.4%). These results indicate the benefit of providing etiologic treatment to young people with chronic infection. This recommendation would have a major health impact both in primary prevention, preventing further cases of congenital Chagas disease, and in secondary prevention, reducing the risks of morbidity and mortality that can cause infection.

## Introduction

The most common transmission routes of Chagas disease are vectorial, transfusional and congenital, but other routes described include oral, organ transplants, needle sharing among injecting drug users and accidents.

In recent years, significant progress has been made in the fight against triatomines, which, added to the controls implemented by blood banks, drastically reduced infections by *Trypanosoma cruzi* through the vectorial and transfusional routes [1].

Given the current migrations from rural areas to urban centers, this way of infection may be encountered may occur in regions considered of low or very low endemicity and even in countries where the vector is not found [2]–[5].

Currently, vertical transmission is considered as the source that generates the highest number of new acute infection cases [6]. However, less than 20% of children born with congenital infection are diagnosed early, due to difficulties in the health system, which fails to perform an adequate follow-up of the newborn [7], [8]. As most infected children have no symptoms, the mothers feel no need for medical intervention, to which one must add the economic difficulties of traveling to the health center. The importance of early detection lies in the possibility of receiving trypanocidal therapy, which is highly effective at this stage of the infection [9]–[11].

The present study is based on a preliminary observation, with a limited number of cases, where it is hypothesized that trypanocidal therapy has beneficial effects in the prevention of congenital transmission of *T. cruzi* [12].

In chronically infected adult patients, antiparasitic treatment was recently reconsidered as useful on different levels of prevention (i.e., primary prevention by interruption of the transmission cycle, secondary prevention by preventing development of the disease, tertiary prevention by preventing progression of the disease) [13]. The trypanocide treatment in pregnant women is not indicated due to lack of evidence of complete safety for potentially teratogenic effects. Although recently were reported cases where trypanocide treatment had to be prescribed by the last trimester of pregnancy. These prescriptions were administered during special situation with risk of life of the mother ongoing an acute phase of *T. cruzi* infection [14], [15].

The supposedly lower efficiency of the etiological treatment in adults compared with children is a consequence of the limited evidence of the efficacy of trypanocidal therapy in producing complete deparasitation. Evaluating this treatment in chronically infected adults requires a very prolonged follow-up due to the slow progression of the disease, serology reactivity even many years after treatment and the lack of sufficiently sensitive parasitological methods.

To date, the cure criterion is the continued absence of anti-*T. cruzi* antibodies evaluated by serology, whereas the treatment failure criterion is the detection of parasitemia after treatment [13].

In order to corroborate the hypothesis of efficacy of the treatment in interrupting the transmission of congenital Chagas disease, the following objective was proposed: to evaluate the effect of the specific antiparasitic treatment in the prevention of congenital Chagas disease. We have analyzed also the serological and clinical evolution of mothers chronically infected with *T. cruzi* treated and untreated with trypanocidal drugs.

## Methods

### Design

We performed a mainly retrospective multicenter, observational cohort study, although it included the information recorded during the development of this project (May 2012 – May 2013).

The cohorts were made up of women-mothers infected with *T. cruzi*, with epidemiological, serological and clinical follow-up, selected by convenience sampling among patients attending the centers involved in this project. A cohort of women had received trypanocidal treatment before pregnancy and the other cohort had not received treatment (control group). We studied the children of these mothers to detect congenital infection.

We analyzed all available information in the medical records of women infected with *T. cruzi* who were under the coverage area of the participating centers in Argentina: Centro de Investigaciones sobre Endemias Nacionales (CIEN) - Facultad de Bioquímica y Ciencias Biológicas- Universidad Nacional del Litoral. Santa Fe; Centro Nacional de Diagnóstico e Investigaciones Endemo-epidemicas, Administración Nacional de Laboratorios e Institutos de Salud (ANLIS), Buenos Aires, Argentina; and the Instituto Nacional de Parasitología (INP), “Dr Mario Fatala Chaben”, Administración Nacional de Laboratorios e Institutos de Salud (ANLIS) Malbrán, Buenos Aires, Argentina.

### Study sample

For assessing the objective, the unit of analysis was the clinical record of the “infected mother with *T. cruzi* in chronic phase-biological child” pair.

The inclusion criteria were:

- for the women: a) treated or untreated women with trypanocidal drugs who at the start of the follow-up had 2 or more reactive serological tests for infection with *T. cruzi*; b) socio-epidemiological information: date of birth; c) trypanocidal treatment information (if the case): date, dose, schedule and grade of completion.
- for the biological children of women: a) confirmed diagnosis of absence or presence of infection with *T. cruzi*, diagnosed by parasitological methods before 10 months age and/or serological methods after 9 months of age; b) socio-epidemiological information: date of birth, places where the child lived before diagnosis; c) healthcare information: whether or not he/she had received transfusions, dates and results of the first parasitological and/or serological tests that confirmed status of infection.

Regarding trypanocidal treatment, it was considered a complete treatment with benznidazole in a dose of 5 mg/kg/day administered in two times a day, during 30–60 days, or with nifurtimox in a dose of 10 mg/kg/day administered in two times a day, during 30–60 days. Tolerance to treatment was considered: i) good, if the patient had no side effects or were very slight, ii) regular, if the patient required symptomatic treatment for side effects but didn't interrupt taking trypanocidal drugs, and iii) bad, if due to side effects it was necessary to interrupt treatment.

Women and child were only recruited if both fulfilled the inclusion criteria.

To assess serological and clinical evolution of mothers, we analyzed only those women who, fulfilling the inclusion criteria mentioned above had a serological follow-up equal to or greater than 8 years.

Serology included the following determinations for the detection of anti-*T. cruzi* antibodies: Indirect hemagglutination (IHA), indirect immunofluorescence (IIF) and enzyme immunoassay (ELISA), performed in each center with internal quality control. The centers take part of the Quality Control Program through the National Network of Laboratories which regularly submit to external quality controls performed by the National Institute of Parasitology. All results of tests obtained as secondary data from medical records or performed during the project and informed as primary were under the same quality control procedures.

Clinical controls consisted of clinical examinations and 12-lead electrocardiogram (ECG). The alterations in the ECG that were compatible with chronic Chagas cardiomyopathy (CCC) were the following: Complete Right Bundle Branch Block (RBBB); Left Anterior Fascicular Block (LAFB) in persons under 50 years of age; frequent Ventricular Extrasystoles (fVE); Second-degree Atrioventricular Block (2°AVB); Complete Atrioventricular Block (CAVB) and electrically “silent” areas with no history of ischemic heart disease.

### Data collection

Each center implemented screening through medical record (MR) review of infected women treated and untreated with trypanocidal drugs. Women who had not had children or that wasn't possible to contact to verify this condition were excluded. Women with chronic infection who had become mothers and their children were considered eligible. Upon verification of the existing data of eligible cases, we used the following strategies: a) retrospective: with secondary data if the MR contained all the information required for inclusion; b) retrospective and prospective: if the MR lacked information for considering inclusion, we proceeded to contact the patient to collect such information, and, if necessary, to conduct studies and sampling for laboratory testing. In the last case, we considered both pre-existing secondary data and primary data generated during the study. Every pair mother-child which had all information considered in the inclusion criteria were “included or recruited”. In case women had more than one child, every child that fulfilled inclusion criteria was considered for a different pair. There were cases of women treated that had children before and after trypanocidal treatment, and so formed part of pairs in both groups.

The information was included in a specifically tailored form with an encrypted identification number to protect the patient's identity. In the mothers' form, the following variables were considered: a) life history of the mother (genealogical data, migration history, medical history, obstetric history): b) infection with *T. cruzi* (way of discovery, serological tests, follow-up studies of infection); c) etiological treatment (whether she received it or not, date, type of drug, dose and time, tolerance level, post-treatment controls using serology with quality control); d) clinical course (clinical and electrocardiography examination). In the children's form variables were collected on: a) life history of the child (genealogical data, migration, transfusion and living conditions history, period of gestation and birth, medical history at birth); b) infection by *T. cruzi* (age at diagnosis, serological and/or parasitological tests).

The information was gathered always by the same specifically trained medical and/or biochemistry professionals, as appropriate.

Subsequently, each paper form with all the information of the mother and her child was uploaded to an on-line database generated “ad hoc”.

### Data analysis

For data analysis, we used the STATA software, version 9.0.

To identify potential confounders, we analyzed distribution in both treated and not treated women, of variables such as maternal age, migration history, socioeconomic status, and level of education collected as epidemiological data from the MR.

The comparison of proportions was performed using a Fisher exact test or χ^2^ as appropriate, and for comparing the means, we used Student's t test or the Bonferroni test. The difference in incidence of congenital Chagas disease in children of mothers treated and untreated was analyzed using the point estimate of relative risk and its confidence interval. The likelihood of negative seroconversion during the follow-up of mothers, according to whether they had received or not specific anti-*T. cruzi* treatment, was evaluated using Kaplan-Meier life tables or curves. We used the log-rank test for the comparative analysis of the rate of serological negativity: a) treated and untreated mothers, and b) by age group of women who received treatment.

### Ethics statement

This project was evaluated and approved by the Ethics Committee of the Faculty of Biochemistry and Biological Sciences at the Litoral National University in its meeting held on November 10, 2011, City of Santa Fe, Santa Fe Province. The participants who were examined and sampled for laboratory studies signed an informed consent to participate in the study. In case participants were minors, parents/guardians provided informed written consent on behalf of their child taking part of the study.

## Results

**Figure 1:**
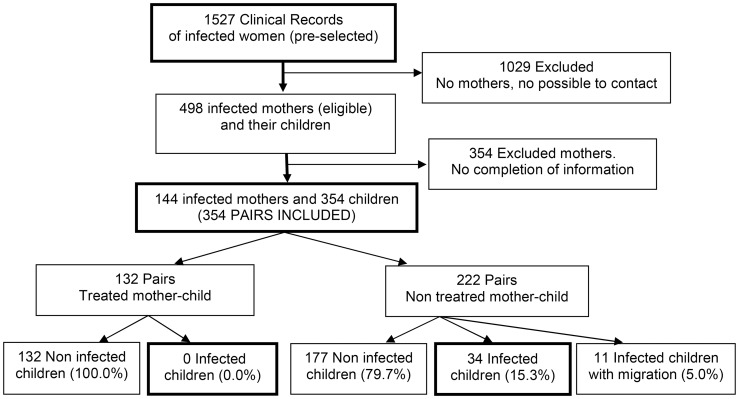
Flowchart of the study.

One thousand and 527 clinical records of infected women were screened, of which 144 mothers were included and made up 354 pairs with their biological children. Reasons for exclusion were: a) the woman did not had any child, b) it wasn't possible to establish contact with the woman to figure out whether she had or not any child and/or to complete information of transfusions and residence of the child, c) the woman had children but they didn't have information on the child's diagnosis and they did not attend to the center to test the child (one or several). Of the included pairs, 132 were made-up with women who had received trypanocidal treatment prior to being mothers and 222 pairs with women who were not treated (Figure 1).

**Figure 2:**
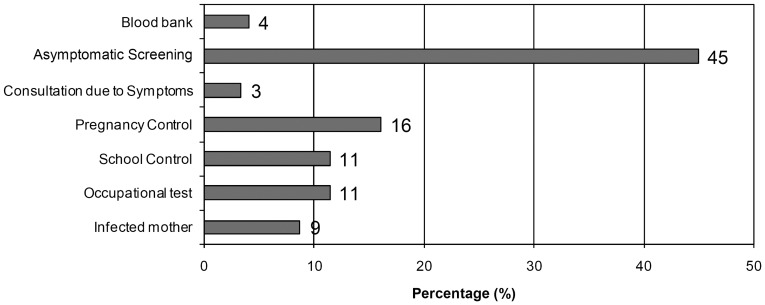
Method for detection of *T. cruzi* infection in 144 mothers.

The geographical origin of the 144 mothers enrolled was as follows: 85% from Argentina; 10% from Bolivia, 4% from Paraguay, and 1% from Uruguay (Table 1). Of the Argentine women, 33% were born and/or lived until 15 years in areas of high or moderate risk for vectorial transmission and 52% lived in houses of precarious conditions, but at the moment of birth of their children only 8% live in those places and 92% lived in areas of low risk or without risk (non-endemic) areas for vectorial transmission and in houses with urban characteristics. No significance difference was observed between the groups of treated and non-treated women. The probable route of infection in these women were: 66% (95/144) was born and/or lived in area of high risk of vectorial infection, 23% (33/144) received transfusions and 31% (45/144) had a history of a mother infected with *T. cruzi.* Some of them had more than one antecedent. Figure 2 shows how the discovery of the infection with *T. cruzi* occurred in the mothers studied.

**Table 1:** Sociodemographic description and exposure of 144 women treated and untreated with trypanocidal drug.

Variable	Categories	Total Women	Treated Women	Non-treated Women
		N (%) ^(a)^	N (%) ^(a)^	N (%) ^(a)^
Total Population		144 (100.0)	88 (100.0)	56 (100.0)
Nationality	Argentine	122 (84.6)	70 (79.5)	52 (92.7)
	Other	22 (15.4)	18 (20.5)	4 (7.3)
Province of origin (birth) according to risk of vectorial transmission ^(b)^	Moderate-high	40 (32.7)	24 (34.3)	16 (30.8)
	Low	63 (51.6)	34 (48.6)	29 (55.8)
	Without risk	19 (15.9)	12 (17.1)	7 (13.5)
Current province of residence according to risk of vectorial transmission ^(b)^ *	Moderate-high	11 (7.7)	11 (12.6)	0 (0.0)
	Low	70 (49.0)	37 (42.5)	33 (58.9)
	Without risk	62 (43.4)	39 (44.8)	23 (41.1)
Type of dwelling <15 years*	Rural	63 (52.1)	37 (50.0)	26 (55.3)
	Urban	58 (47.9)	37 (50.0)	21 (44.7)
Current type of dwelling*	Rural	12 (8.4)	11 (12.6)	1 (1.8)
	Urban	131 (91.6)	76 (87.4)	55 (98.2)
Received transfusions*	Yes	30 (25.2)	15 (20.8)	15 (31.9)
	No	89 (74.8)	57 (79.2)	32 (68.1)

a) Comparison of frequencies by Chi^2^ test or Fisher test. Comparison of means by Student's T test or Bonferroni test.

b) Only for Argentinian provinces, according to the denomination used by the Programa Nacional de Chagas (last viewed 29/10/13).

*Variable with N lower than total population.

Regarding etiological treatment 72 women were treated with benznidazole, 16 women treated with nifurtimox, and 56 women did not receive treatment. Treatment with benznidazole consisted of a media dose of 5.16 mg/kg/day (DS 0.47, range 4.13–6.35) during 15–66 days (media 45±14). There were five cases of less than 30 days of treatment (15, 17, 25, 27 and 28 days). Variation in time was due to different recommendations considered in national protocols in different periods and interruptions. Treatment with nifurtimox was with a media dose of 10 mg/kg/day (DS 0.73, range 8–12) administered for 14–90 days (media 47±18). Variation in days occurred due to different medical criteria and/or interruption. Tolerance was good for 86.3% (57/72) of women treated with benznidazol and 62.5% (10/16) of those treated with nifurtimox. One woman treated with nifurtimox (6.2%) and one treated with benznidazole (1.4%) had bad tolerance, which forced them to discontinue the medication. Side effects for treatment with benznidazole were rashes, pruritus, and/or edema 20.8% (15/72). Among women treated with nifurtimox, the most common side effects were gastrointestinal disorder, 37.5% (6/16).

The 56 non-treated women had 222 children, of which 45 (20.3%) were confirmed infected with *T. cruzi*. Of these, 11 children had a history of having lived in regions where they were likely to be infected through vectorial transmission (migration history); therefore, there is no certainty that the infection route was congenital transmission. The remaining 34 children (15.3%) presented positive maternal serology as the only antecedent, not having received blood transfusions or resided in areas at risk of vectorial infection. From the group of 34 congenitally infected children, in five cases confirmatory diagnosis was made by visualization of the parasite through xenodiagnosis or strout method before their first year, and the rest were diagnosed by positive serology after 10 months of age. The average age of diagnosis was 5.8 years (SD 5.6, range 0.1–19.9 years).

Of the 132 children born to the 88 women who had been treated before pregnancy, none had infection with *T. cruzi*. Five of them came from two mothers with incomplete treatment. The diagnosis was made at an average age of 4.8 years (SD 4.4; range 0.8 to 20.0). If we assumed one positive case in the treated group, the risk of the occurrence of congenital transmission in treated mothers was 25 times lower compared to those untreated before pregnancy (Relative Risk, RR  = 0.04, CI:95%: 0.012 to 0.166; p<0.05).

The gestational age and mode of delivery did not differ between the children of treated and untreated mothers, nor between healthy newborns and newborns with congenital infection (p>0.05, Fisher exact test). The birth weight of the children was similar regarding treatment in mothers and status of infection in the newborn (Student's t-test, p>.05) (Table 2).

**Table 2:** Epidemiological data.

		Total	Average weight (mg) *	Gestational Age n (%)			Type of delivery n (%)		
				Term	Pre-term (%)	no data	Vaginal	Caesarean section	no data
Children of treated mothers	Infected *T. cruzi*	0	-	-	-	-	-	-	-
	Uninfected *T. cruzi*	132	3387	98 (74)	4 (3)	30 (27)	74 (56)	27 (20)	31 (24)
Children of non-treated mothers	Infected *T. cruzi*	45	3164	40 (89)	5 (11)	0 (0)	38 (85)	7 (15)	0 (0)
	Uninfected *T. cruzi*	177	3389	159 (90)	6 (3)	12 (7)	122 (69)	43 (24)	12 (7)

Birth weight, gestational age and type of delivery of the children with or without infection by *Trypanosoma cruzi* born to infected women treated and untreated with trypanocidal. Argentina.

Fisher's exact test, p>0.05.

*Student's t test, p>0.05.

The average age of mothers at childbirth was 26.6±6.5 years and 25.4±6.2 years for treated and untreated mothers, respectively. We found no differences when comparing the age of mothers at childbirth, by parity, the occurrence of congenital infection, and having received treatment (Bonferroni p>0.05).

Table 3 describes the population of studied mothers grouped by age and time of follow-up. For the analysis of the serological and clinical evolution (SCE) of mothers untreated and treated with trypanocidal drugs, we selected 117 mothers (71 mothers treated, and 46 mothers untreated) out of 144 that made up the pairs, with a follow-up equal to or greater than 8 years.

**Table 3:** Distribution of 117 women with chronic infection of *T. cruzi,* treated and untreated with nifurtimox or benznidazole, by age group and time of follow-up equal to or greater than 8 years, Argentina.

Age Group *	TREATED				NON-TREATED
	N according to Drug		Follow-up Time (years ± Sd)	N	Follow-up Time (years ± Sd)
	Nifurtimox	Beznidazol			
≤15	2	23	16.3±5.8	0	-
16–25	2	16	16.1±9.2	11	22.8±7
26–35	9	13	18.5±10	25	16.5±8.2
>35	2	4	18.1±6.1	10	19.4±10.1
Total	15	56	17.1±8.1	46	18.6±8.6

The following groups were formed according to age and treatment: A) 25 women who were treated up to 15 years of age (23 treated with benznidazole and 2 with nifurtimox); B) 46 women treated after 15 years of age (33 with benznidazole and 13 with nifurtimox); C) 46 untreated women over 15 years old.

The average ages at the time of treatment or start of follow-up were: group A) 9.97±2.74 years; B) 27.9±6.7 years; C), 29.2±6.2 years.

The average follow-up times were 16.3±5.8, 17.5±9.2 and 18.6±8.6 years for groups A, B and C, respectively.

### Serological evolution

#### Group A

Sixteen out 25 women (64%) had negative serology after an average time of 17.5 years; 4 out of 25 (16%) had a discordant final serology status (weak reactivity at the cut-off for any of the serological tests and non-reactivity in another), with an average follow-up of 13.3 years. The remaining 20% (5/25) of treated women remained with reactive serology until the last control and were followed for 14.7 years on average.

#### Group B

Fifteen out of 46 (32.6%) had negative serology after an average time of 24.2 years; 6/46 (13%) had a discordant final serology status, with average follow-up of 23.6 years. 54.4% (25/46) of the treated women remained with reactive serology until the last control, followed for 12 years on average.

#### Group C

2/46 (4.4%) showed fluctuations during the follow-up, with discordant final serology, while 44/46 (95.6%) remained with reactive serology without significant changes in serologic titers for an average 18.6 years.

**Figure 3:**
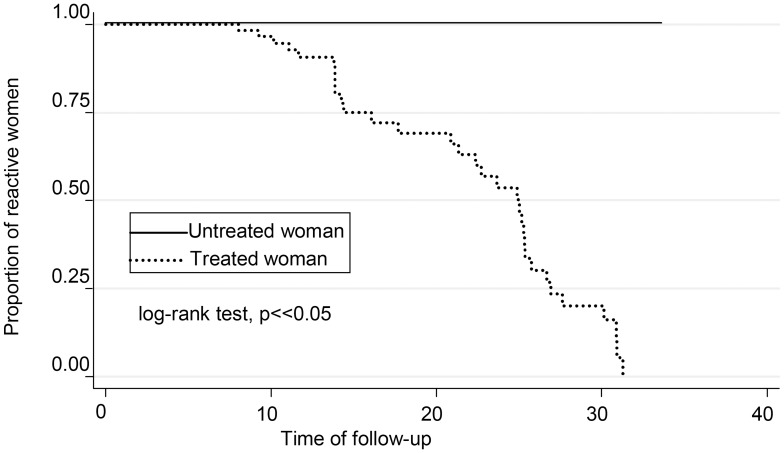
Kaplan-Meier curve showing serological reactivity rate during follow-up among 117 treated and untreated women.

**Figure 4:**
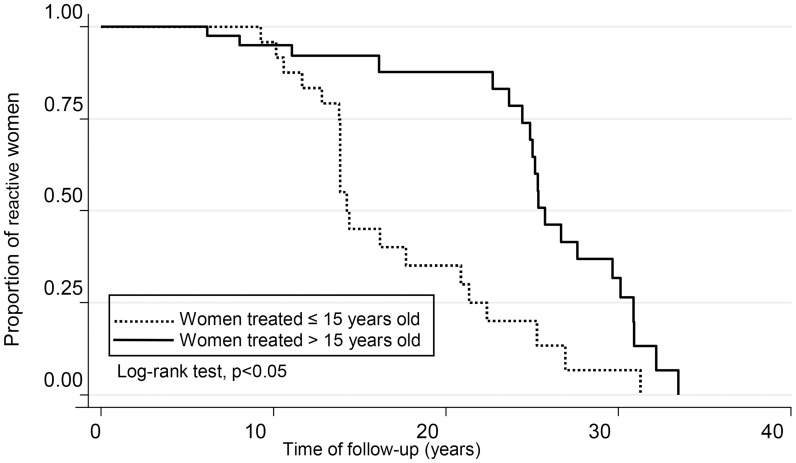
Kaplan-Meier curve showing serological reactivity rate by age during follow-up in 71 treated women.

Sero-negativization was observed in treated women and not in untreated ones (Figure 3). This phenomenon was observed after 10 years of follow-up (p = 0.0001 log-rank test). There were differences when analyzing the probability of negative seroconversion according to the age at which the mother received treatment (group A vs. group B). Women who were treated before or at 15 years of age presented earlier negativization, compared to those treated at ages higher than 15 years. For those women treated at age of 15 years old or earlier (A), they reached a 50% probability of seronegativization at 15 years of follow-up, meanwhile those treated between 16 to 45 (B) reached 50% probability of seroconversion only at 26 years of follow-up (p = 0.0004, log-rank test) (Figure 4).

### Clinical progression

The ECG alterations compatible with CCC presented by the mothers with follow-up are shown in Table 4. No alterations suggestive of CCC were observed in treated women of group A (0/25), and in group B only one woman modified her clinical status 1/46 (2.2%), developing LAFB and fVE during follow-up at the age of 48 years. Two other women (01-144-00 and 02-048-00) that presented ECG alterations during the study had also associated pathologies and therefore it was no possible to establish causality of these alterations to Chagas disease.

**Table 4:** Evolution of electrocardiographic alterations compatible with chronic Chagas cardiomyopathy (CCC) that appeared during follow-up in women treated and untreated with trypanocides, Argentina.

	TREATED					
	Initial ECG			Final ECG		
ID	Age (years)	Alt ECG	Assoc Patolog	Age (years) *	Alt ECG	Assoc Patolog
01-144-00	30	Normal	No	54	LAFB	AHT - age
01-351-00	25	Normal	No	48	LAFB + fVE	No
02-048-00	34	Normal	No	40	Sinus bradycardia	WPW syndrome †

	UNTREATED					
	Initial ECG			Final ECG		
ID	Age (years)	Alt ECG	Assoc Patolog	Age (years) *	Alt ECG	Assoc Patolog
01-213-00	19	Normal	No	42	fVE	No
01-262-00	30	Normal	No	49	LAFB	No
01-356-00	23	Normal	No	45	RBBB	No
01-362-00	31	Normal	No	40	LAFB	No
01-365-00	25	Normal	No	48	RBBB + LAFB	No
01-366-00	29	Normal	No	37	LAFB	No
02-031-00	37	Normal	No	42	LAFB	No

*Age at start of ECG alterations.

† Wolf-Parkinson-White Syndrome.

RBBB: Complete Right Bundle-Branch Block.

LAFB: Left Anterior Fascicular Block.

fVE: Frequent Ventricular Extrasystoles.

AHT: Arterial Hypertension.

Among the women who remained untreated (group C), 7/46 (15.2%) became ill with alterations attributable to CCC.

There were significant differences in clinical progression between treated and untreated women of ages older than 15 years (p<0.05, Fisher exact test; Relative Risk, RR  = 0.14, CI:95%: 0.02 to 0.80).

## Discussion

There was no case of congenital transmission among the children born to mothers that received treatment before pregnancy, whereas in the group of children from non treated mothers there were 34 cases (15.3%). This finding supports the hypothesis of a protective effect of trypanocidal treatment before pregnancy for preventing congenital transmission, and a risk for transmission of *T. cruzi* in newborn from treated mothers of 25 times lower (assuming one case of transmission in the treated group). The rate of congenital transmission found in the non treated group is high compared to the average rate of 4.7% (CI95%; 3.9 to 5.6%) [5], although other studies have found higher numbers; for example, 11% by Rissio AM and colleagues [16] in a non endemic area and 17% by Sosa-Estani S and colleagues [17] in an endemic area. However this endemic area (Gran Chaco Region) was sprayed, it had a recent risk for vectorial transmission which might increase congenital transmission rate since these mothers have probably higher parasite burden due to probable repetitive infections [17]–[20]. Although the residence area of the study population was urban areas or areas under surveillance, our study did not assessed parasitic load in mothers to explain this potential phenomenon where the risk of re-infections could determine high parasitemias. These variations in vertical transmission rates could be attributed to differences in the methodology and the techniques used for diagnosis and when the control of the children is done. Other factors that may have an impact are the parasite load in the mother and/or the strain of *T. cruzi*, and the immunological and/or nutritional status of pregnant women, although none has been conclusively demonstrated [19].

Besides these considerations, we could have overestimated the detected cases of congenital Chagas due to a possible “memory” bias in infected subjects that had a late diagnosis (average age of children at diagnosis: 5.8 years). Although we excluded those who had traveled or lived in areas at risk of vectorial infection, we could have included some cases of vectorial infection or of infection by a route other than congenital, as the information was obtained from the answers given by the mothers in personal interviews. This method of data collection was applied to the entire study population.

While not all women infected with *T. cruzi* transmitted the infection to their children, the cases of congenital Chagas disease only occurred in infants born to mothers who had not received specific deparasitation treatment. It was shown that the relative risk of transmitting the infection is 25 times higher for untreated mothers than for those who received treatment before pregnancy. It was thus showed that etiological treatment is useful to prevent congenital infection by *T. cruzi*, corroborating the results of a preliminary investigation [12].

The scheme of treatment (drug, dose, duration) that had been applied to the treated mothers was according the official guidelines of care of patients infected with *T. cruzi* in Argentina [21]. These guidelines recommend treatment by 60 days but, consider an acceptable scheme 30 days of treatment when any justified reason does not allow completing the treatment.

No differences were found between children born infected and uninfected regarding birth weight, gestational age and mode of delivery. The mother's age at childbirth had also no influence. This corroborates the lack of a relevant clinical expression of symptoms and/or signs that characterize most newborns with congenital Chagas disease, and the vital need to actively look for them.

The children with congenital infection who were diagnosed after one year of age (average age of 5.8 years) confirm the deficiency of the health system in making an early diagnosis.

The need to expand the coverage of early diagnosis is also shown by the 16% of mothers who became aware of their infected condition during pregnancy, and the 11% with serological controls at school age.

When assessing the evolution of conventional serology titers in the mothers studied, we observed negativization only in those who had received trypanocidal treatment, in a 43.7% (31/71). This percentage could be enlarged in the coming years by the 14.1% (10/71) with discordant serology at the last control. This presumption is based on the fact that those who became negative had a slightly higher average follow-up time.

The age at which the infected patient received treatment is important, as significant differences in the probability and time of negative seroconversion were observed between women treated before and after 15 years of age. The negative seroconversion rates observed when treatment was administered at a later age are also noteworthy, even though more time was required.

In conjunction with these serological results, we observed a better clinical course in women who received trypanocidal treatment compared with those who were not treated. These results are consistent with those observed by other investigations or reviews and by our group [22]–[27].

There were some limitations and potential biases. There could be a selection bias because the population sample was selected by convenience sampling not randomly or probabilistically, as was mentioned above. The measurement of the degree of adherence and compliance with the treatment performed on an outpatient basis was not recorded by direct observation, using instead what was expressed by the patient and was reported in the MR. There might have been potential recall biases, but the answers given by the patient in the personal interview were considered acceptable, as was the criterion of the professional that recorded the information.

Currently, the specific antiparasitic treatment must be mandatorily administered in all cases of acute infection because its effectiveness at this stage is close to 100%; also in cases of chronically infected persons less than 19 years of age and in cases of reactivation by immunosuppression at any age [21]. Trypanocidal treatment could also be offered to adults with chronic infection by *T. cruzi* [21].

These results add evidence about the benefit of providing etiological treatment to young people with chronic infection. Since the treatment proved its effectiveness in reducing the risk of mother to child transmission, it would be an efficient practice for the reduction or complete prevention of new cases of congenital infection with *T. cruzi* in both rural and urban centers.

Furthermore its importance as a form of primary prevention to reduce or prevent new cases of congenital Chagas disease, the recommendation of indicating trypanocidal treatment would also serve as a secondary prevention mechanism due to the protective effect observed during the clinical course. By eliminating or reducing at least 43.7% of the parasite load, the risks of morbidity and mortality of the infection would decrease significantly. This would lead to a decrease in the burden of the disease by reducing public health costs and preventing the economic and social impact produced by the deterioration of the quality of life of infected people; the expense in heart disease drugs, pacemakers, defibrillators, transplants, etc. caused by the symptomatic treatment of the chronic phase would decrease too [28]–[29].

Despite the promise offered by these results, it would be useful to extend the study population. Participation of other institutions that take care of patients chronically infected with *T. cruzi* would help to define with more certainty the most appropriate therapy and prophylaxis for these patients.

## Supporting Information

Checklist S1 **STROBE checklist.**
(DOC)
