# Supplementary material for: Trypanocide Treatment of Women Infected with Trypanosoma cruzi and Its Effect on Preventing Congenital Chagas
Source: PLoS Negl Trop Dis. 2014 Nov 20;8(11):e3312. doi: 10.1371/journal.pntd.0003312 (PMC4239005; doi:10.1371/journal.pntd.0003312)
Supplement: Checklist S1 — STROBE checklist. (DOC) [file pntd.0003312.s001.doc]

STROBE Statement—checklist of items that should be included in reports of observational studies

|  | Item No | Recommendation |
| --- | --- | --- |
| **Title and abstract** | 1 | (*a*) Indicate the study’s design with a commonly used term in the title or the abstract  We conducted a multicenter, observational study on a cohort of mothers infected with Trypanosoma cruzi, with and without trypanocidal treatment before pregnancy. Their children were studied to detect congenital infection. |
|  |  | (*b*) Provide in the abstract an informative and balanced summary of what was done and what was found.  We evaluated the efficacy of trypanocidal therapy in preventing congenital Chagas disease and compared the clinical and serological changes between treated and untreated mothers. From medical records of infected women who had received or not trypanocidal treatment, we evaluated their children to detect congenital infection. Among 222 children of untreated mothers, we detected 34 cases of congenital transmission. The 132 children of previously treated women were born without infection. |
| Introduction | | |
| Background/rationale | 2 | Explain the scientific background and rationale for the investigation being reported  With the control of the vectorial and transfusional routes of infection with Trypanosoma cruzi, congenital transmission has become an important source of new cases. Also due to migrations from rural areas to urban centers, this way of contracting the infection may occur in regions considered of low or very low endemicity and even in countries where the vector is not found.  In Argentina less than 20% of children born with congenital infection are diagnosed early, due to difficulties in the health system, which fails to perform an adequate follow-up of the newborn.  A previous observation had shone that from 32 children born to mothers treated when they were 4-16 years old, no congenital transmission of T.cruzi occurred. From this it was hypothesized that trypanocidal therapy has beneficial effects in the prevention of congenital transmission of T. cruzi. |
| Objectives | 3 | State specific objectives, including any prespecified hypotheses  The objectives of the study were:  1º Evaluate occurrence of congenital transmission of T.cruzi in children of infected women who had received or not tripanocidal treatment before pregnancy.  2º Evaluate serological and clinical evolution of treated and untreated women.  The hypothesis of the study was that tripanocidal treatment in women before pregnancy has a preventive effect for congenital transmission of T.cruzi. |
| Methods | | |
| Study design | 4 | Present key elements of study design early in the paper  We performed a mainly retrospective multicenter, observational cohort study, although it included the information recorded during the development of this project (May 2012 - May 2013). |
| Setting | 5 | Describe the setting, locations, and relevant dates, including periods of recruitment, exposure, follow-up, and data collection  The cohorts were made ​​up of women-mothers infected with T. cruzi, with epidemiological, serological and clinical follow-up, selected by convenience sampling among patients attending the centers involved in this project.  Each center implemented screening through medical record (MR) review of infected women. When the information needed was incomplete or not available in the MR we contacted the women to ask about it. Women who had not had children or that wasn’t possible to contact to verify this condition were excluded. Women with chronic infection who had became mothers and their children were considered eligible. Upon verification of the existing data of eligible cases, we used the following strategies: a) retrospective: with secondary data if the MR contained all the information required for inclusion; b) retrospective and prospective: if the MR lacked information for considering inclusion, we proceeded to contact the patient to collect such information, and, if necessary, to conduct studies and sampling for laboratory testing. In the last case, we considered both pre-existing secondary data and primary data generated during the study.  Women included were from Argentina (85%), Bolivia (10%), Paraguay (4%) and Uruguay (1%), but all lived in Argentina at the moment of the study. At the moment of birth of their child almost all (92%) lived in areas of low risk or without risk (non-endemic) areas for vectorial transmission and in houses with urban characteristics. No significance difference was observed between the groups of treated and non treated women. |
| Participants | 6 | (*a*) *Cohort study*—Give the eligibility criteria, and the sources and methods of selection of participants. Describe methods of follow-up  *Case-control study*—Give the eligibility criteria, and the sources and methods of case ascertainment and control selection. Give the rationale for the choice of cases and controls  *Cross-sectional study*—Give the eligibility criteria, and the sources and methods of selection of participants  The inclusion criteria were:  -for the women: a) treated or untreated women with trypanocidal drugs who at the start of the follow-up had 2 or more reactive serological tests for infection with T.cruzi; b) socio-epidemiological information: date of birth; c) trypanocidal treatment information (if the case): date, dose, schedule and grade of completion.  -for the biological children of women: a) confirmed diagnosis of absence or presence of infection with T.cruzi, diagnosed by parasitological methods before 10 months of age and/or serological methods after 9 months of age; b) socio-epidemiological information: date of birth, places where the child lived before diagnosis; c) healthcare information: whether or not he/she had received transfusions, dates and results of parasitological and/or serological tests.  Every pair mother-child which had all information considered in the inclusion criteria were “included or recruited”. |
|  |  | (*b*) *Cohort study*—For matched studies, give matching criteria and number of exposed and unexposed  *Case-control study*—For matched studies, give matching criteria and the number of controls per case |
| Variables | 7 | Clearly define all outcomes, exposures, predictors, potential confounders, and effect modifiers. Give diagnostic criteria, if applicable  For the mothers, the following data was collected: a) life history of the mother: genealogical data, migration history, medical history, obstetric history; b) infection with T. cruzi: way of discovery, serological tests, follow-up studies of infection; c) etiological treatment: whether she received it or not, date, type of drug, dose and time, tolerance level, post-treatment controls using serology with quality control; d) clinical course: clinical and electrocardiography examination. For the children the following data was collected: a) life history of the child: genealogical data, migration, transfusion and living conditions history, period of gestation and birth, medical history at birth; b) infection by T. cruzi (age at diagnosis, serological and/or parasitological tests). |
| Data sources/ measurement | 8* | For each variable of interest, give sources of data and details of methods of assessment (measurement). Describe comparability of assessment methods if there is more than one group  Each center implemented screening through medical record (MR) review of infected women. When the information needed was incomplete or not available in the MR we contacted the women to ask about it. Information on serological tests, electrocardiographic examination and treatment was only considered from MR or other source in which a professional had verified the condition. Data were collected from MR using a specific data form for this project, and all the information of the mother and her child was uploaded to an on-line database generated "ad hoc".  Status of infection in mothers and children older than 10 months was evaluated through two or more serological test. Serology included determinations for the detection of anti-T. cruzi antibodies by the following techniques: Indirect hemagglutination (IHA), indirect immunofluorescence (IIF) and enzyme immunoassay (ELISA), performed in each center with internal quality control. To determine the status of infection in children before 10 month of age it was considered parasitological methods as Strout, microhematocrit, or “INP micromethod” (validated technique). The centers take part of the Quality Control Program through the National Network of Laboratories which regularly submits to external quality controls performed by the National Institute of Parasitology. All results of tests obtained as secondary data from medical records or performed during the project and informed as primary were under the same quality control procedures.  Clinical controls consisted of clinical examinations and 12-lead electrocardiogram (ECG). The alterations in the ECG that were compatible with chronic Chagas cardiomyopathy (CCC) were the following: Complete Right Bundle Branch Block (RBBB); Left Anterior Fascicular Block (LAFB) in persons under 50 years of age; frequent Ventricular Extrasystoles (fVE); Second-degree Atrioventricular Block (2°AVB); Complete Atrioventricular Block (CAVB) and electrically "silent" areas with no history of ischemic heart disease. |
| Bias | 9 | Describe any efforts to address potential sources of bias  Regarding status of infection, clinical condition and treatment, we only considered information evaluated by professional and stated on MR or other report to avoid incorrect information given by mothers due to memory bias or misunderstanding.  In children, to consider any probability of infection by other transmission route than congenital, we obtained information on transfusion and history of residence or trips to areas of some risk for contact with vector, as well as building characteristics of houses. |
| Study size | 10 | Explain how the study size was arrived at  We performed a convenience sampling among patients attending the centers involved in this project. All patients who fulfilled the inclusion criteria were considered. |
| Quantitative variables | 11 | Explain how quantitative variables were handled in the analyses. If applicable, describe which groupings were chosen and why  Data for quantitative variables was collected as original continuous measurements. Variables of time as age of treatment, age of delivery, age of dganosis, etc. were calculated considering the date of birth and the event of interest. For the age of treatment we generated a categorized variable considering if treatment had been received before or after 15 years of age, as this is the age for which regulation stablished mandatory treatment.  All other quantitative variables were analysed as continuous measurements. |
| Statistical methods | 12 | (*a*) Describe all statistical methods, including those used to control for confounding  The comparison of proportions was performed using a Fisher exact test or χ2 as appropriate, and for comparing the means, we used Student's t test or the Bonferroni test. The difference in incidence of congenital Chagas infection in children of mothers treated and untreated was analyzed using the point estimate of relative risk and its confidence interval. The likelihood of negative seroconversion during the follow-up of mothers, according to whether they had received or not specific anti-T. cruzi treatment, was evaluated using Kaplan-Meier life tables or curves. We used the log-rank test for the comparative analysis of the rate of serological negativity: a) treated and untreated mothers, and b) by age group of women who received treatment. |
|  |  | (*b*) Describe any methods used to examine subgroups and interactions  For the age of treatment we generated a categorized variable considering if treatment had been received before or after 15 years of age, The likelihood of negative seroconversion during the follow-up of mothers, according to whether they had received or not specific anti-T. cruzi treatment, was evaluated using Kaplan-Meier life tables or curves. We used the log-rank test for the comparative analysis of the rate of serological negativity: a) treated and untreated mothers, and b) by age group of women who received treatment. |
|  |  | (*c*) Explain how missing data were addressed  When the information for a variable wasn´t available for all cases, proportions and statistical estimations between groups were calculated considering the total number of observations for that variable (and not considering the total cases of the study). There was no missing data for the variables of the main outcomes as they were considered inclusion criteria. |
|  |  | (*d*) *Cohort study*—If applicable, explain how loss to follow-up was addressed  *Case-control study*—If applicable, explain how matching of cases and controls was addressed  *Cross-sectional study*—If applicable, describe analytical methods taking account of sampling strategy |
|  |  | (*e*) Describe any sensitivity analyses  No sensitivity analyses was performed |

Continued on next page

| Results | | |
| --- | --- | --- |
| Participants | 13* | (a) Report numbers of individuals at each stage of study—eg numbers potentially eligible, examined for eligibility, confirmed eligible, included in the study, completing follow-up, and analysed  1527 clinical records of infected women were screened, of which 144 mothers were included and made up 354 pairs with their biological children. Reasons for exclusion were: a) the woman didn´t had any child, b) it wasn´t possible to establish contact with the woman to figure out whether had or not any child and/or to complete information of transfusions and residence of the child, c) the woman had children but they didn’t have information on the child’s diagnosis and they didn´t attend to center to test the child (one or several). Of the included pairs, 132 were with women who had received trypanocidal treatment prior to being mothers and 222 pairs with women who were not treated (Figure 1).  **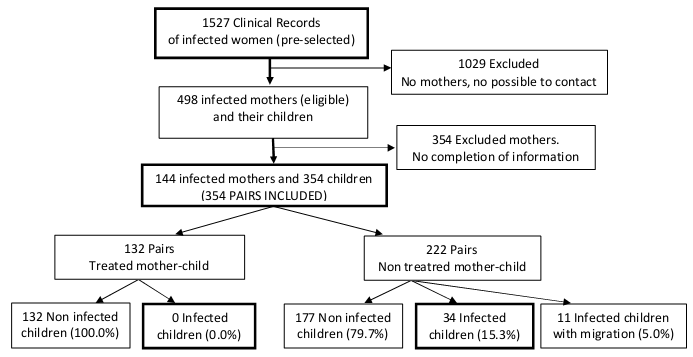** |
|  |  | (b) Give reasons for non-participation at each stage  See bellow (a) |
|  |  | (c) Consider use of a flow diagram  See bellow (a) |
| Descriptive data | 14* | (a) Give characteristics of study participants (eg demographic, clinical, social) and information on exposures and potential confounders  The geographical origin of the 144 mothers enrolled was as follows: 85% from Argentina; 10% from Bolivia, 4% from Paraguay, and 1% from Uruguay (Table 1). Of the Argentine women, 33% were born and/or lived until 15 years in areas of high or moderate risk for vectorial transmission and 52% lived in houses of precarious conditions, but at the moment of birth of their children only 8% live in those places and 92% lived in areas of low risk or without risk (non-endemic) areas for vectorial transmission and in houses with urban characteristics. No significance difference was observed between the groups of treated and non treated women. The probable route of infection in these women were: 66% (95/144) was born and/or lived in area of high risk of vectorial infection, 23% (33/144) received transfusions and 31% (45/144) had a history of a mother infected with T. cruzi. Some of them had more than one antecedent.  The gestational age and mode of delivery did not differ between the children of treated and untreated mothers, nor between healthy newborns and newborns with congenital infection (p> 0.05, Fisher exact test). The birth weight of the children was similar for the variables of the mothers' treatment and newborn infection (Student's t-test, p>.05). (Table 2)  The average age of mothers at childbirth was 26.6 ± 6.5 years and 25.4 ± 6.2 years for treated and untreated mothers, respectively. We found no differences when comparing the age of mothers at childbirth, by parity, the occurrence of congenital infection, and having received treatment (Bonferroni p>0.05). |
|  |  | (b) Indicate number of participants with missing data for each variable of interest  Total sample size of mothers: 144  Variables: N missing value  Mothers  Current province of residence according to risk of vectorial transmission: 1  Type of dwelling <15 years: 23  Current type of dwelling: 1  Received transfusions: 25  Total sample size of Children: 354  Gestational Age: 42  Type of delivery: 43 |
|  |  | (c) *Cohort study*—Summarise follow-up time (eg, average and total amount)  The average follow-up times were:  Group A (treated ≤15 years old): 16.3 ± 5.8 years  Group B (treated >15 years old): 17.5 ± 9.2 years  Group C (untreated): 18.6 ± 8.6 years |
| Outcome data | 15* | *Cohort study*—Report numbers of outcome events or summary measures over time  -Congenital infection:  Children born to treated mothers (before pregnancy): 0/132 (0.0%)  Children born to non-treated mothers: 34/222 (15.3%)  -Serological evolution:  Group A: Sixteen out 25 women (64%) had negative serology after an average time of 17.5 years; 4 out of 25 (16%) had a dubious final serology status (weak reactivity at the cut-off for any of the serological tests and non-reactivity in another), with an average follow-up of 13.3 years. The remaining 20% ​​(5/25) of treated women remained with reactive serology until the last control and were followed for 14.7 years on average.  Group B: Fifteen out of 46 (32.6%) had negative serology after an average time of 24.2 years; 6/46 (13%) had a dubious final serology status, with average follow-up of 23.6 years. 54.4% (25/46) of the treated women remained with reactive serology until the last control, followed for 12 years on average.  Group C: 2/46 (4.4%) showed fluctuations during the follow-up, with discordant final serology, while 44/46 (95.6%) remained with reactive serology without significant changes in serologic titers for an average 18.6 years.  -Clinical evolution:  Group A: No alterations suggestive of CCC were observed (0/25),  Group B: One woman modified her clinical status 1/46 (2.2%), developing LAFB and fVE during follow-up at the age of 48 years.  Group C: 7 out of 46 women (15.2%) became ill with alterations attributable to CCC. |
|  |  | *Case-control study—*Report numbers in each exposure category, or summary measures of exposure |
|  |  | *Cross-sectional study—*Report numbers of outcome events or summary measures |
| Main results | 16 | (*a*) Give unadjusted estimates and, if applicable, confounder-adjusted estimates and their precision (eg, 95% confidence interval). Make clear which confounders were adjusted for and why they were included  From  The 56 non-treated women had 222 children, of which 45 (20.3%) were confirmed infected with T. cruzi. Of these, 11 children had a history of having lived in regions where they were likely to be infected through vectorial transmission (migration history); therefore, there is no certainty that the infection route was congenital transmission. The remaining 34 children (15.3%) presented positive maternal serology as the only antecedent, not having received blood transfusions or resided in areas at risk of vectorial infection.  Of the 132 children born to the 88 women who had been treated before pregnancy, none had infection with T. cruzi. Five of them came from two mothers with incomplete treatment. The diagnosis was made ​​at an average age of 4.8 years (SD 4.4; range 0.8 to 20.0). If we assumed one positive case in the treated group, the risk of the occurrence of congenital transmission in treated mothers would be 25 times lower compared to those untreated before pregnancy (Relative Risk, RR=0.04, CI:95%: 0.012 to 0.166; p<0.05). |
|  |  | (*b*) Report category boundaries when continuous variables were categorized |
|  |  | (*c*) If relevant, consider translating estimates of relative risk into absolute risk for a meaningful time period  See bellow (a) |
| Other analyses | 17 | Report other analyses done—eg analyses of subgroups and interactions, and sensitivity analyses  Sero-negativization was only observed in treated women (groups A and B). This phenomenon was observed after 10 years of follow-up (p=0.0001 log-rank test).There were differences when analyzing the probability of negative seroconversion according to the age at which the mother received treatment (group A vs. group B). Women who were treated before or at 15 years of age presented earlier negativization, compared to those treated at ages higher than 15 years. For those women treated at age of 15 years old or earlier (A), they reached a 50% probability of seronegativization at 15 years of follow-up, meanwhile those treated between 16 to 45 (B) reached 50% probability of seroconversion only at 26 years of follow-up (p=0.0004, log-rank test). |
| Discussion | | |
| Key results | 18 | Summarise key results with reference to study objectives  The primary objective of the study was to evaluate the occurrence of congenital transmission within children of women who had received or not tripanocidal treatment before pregnancy. We evaluated 354 pairs mother-sun, of which 132 had been exposed to treatment and 222 had not received treatment. Within the 132 children born to treated mothers there was no case of congenital infection with T.cruzi (0.0%). Within the 222 children of untreated mothers we found 34 cases in which the only antecedent for the infection was the maternal infection, which means a congenital transmission rate of 15.3%. |
| Limitations | 19 | Discuss limitations of the study, taking into account sources of potential bias or imprecision. Discuss both direction and magnitude of any potential bias  There were some limitations and potential biases. There could be a selection bias because the population sample was selected by convenience sampling not randomly or probabilistically, as was mentioned above. The measurement of the degree of adherence and compliance with the treatment performed on an outpatient basis was not recorded by direct observation, using instead what was expressed by the patient and was reported in the MR. There might have been potential recall biases, but the answers given by the patient in the personal interview were considered acceptable, as was the criterion of the professional that recorded the information.  We could have overestimated the detected cases of congenital Chagas due to a possible "memory" bias in infected subjects that had a late diagnosis (average age of children at diagnosis: 5.8 years). Although we excluded those who travelled or lived in areas at risk of entomological infection, we could have include some cases of vectorial infection or of infection by a route other than congenital, as the information was obtained from the answers given by the mothers in personal interviews. This method of data collection was applied to the entire study population. |
| Interpretation | 20 | Give a cautious overall interpretation of results considering objectives, limitations, multiplicity of analyses, results from similar studies, and other relevant evidence  There was no case of congenital transmission among the children born to mothers that received treatment before pregnancy, whereas in the group of children from non treated mothers there were 34 cases (15.3%). This finding supports the hypothesis of a protective effect of trypanocidal treatment before pregnancy for preventing congenital transmission, and a risk for transmission of T.cruzi in newborn from non treated mothers of 25 times higher (assuming one case of transmission in the treated group). The rate of congenital transmission found in the non treated group is high compared to the average rate of 4.7% (CI95%; 3.9 to 5.6%) [Howard 2013], although other studies have found higher numbers; for example, 11% by De Rissio AM and colleagues [De Rissio 2009] in a non endemic area and 17% by Sosa-Estani S and colleagues [Sosa-Estani 2009] in an endemic area. |
| Generalisability | 21 | Discuss the generalisability (external validity) of the study results  Concerning the transmission rate within untreated mothers we shouldn´t generalize  As the study sample refers to a population that attend to reference centers we should be very careful to generalize, especially concerning the transmission rate.  Concerning the transmission rate within untreated mothers we should not generalize  As the study sample refers to a population that attend to reference centers we should be very careful to generalize, especially concerning the transmission rate.  Regarding the preventive effect of trypanocidal treatment for congenital transmission, despite the promise offered by these results, it would be useful to extend the study population. Participation of other institutions that take care patients chronically infected with T. cruzi would help to define with more certainty the most appropriate therapy and prophylaxis for these patients. |
| Other information | | |
| Funding | 22 | Give the source of funding and the role of the funders for the present study and, if applicable, for the original study on which the present article is based  The study was partially funded by the program “Salud Investiga” of the Ministry of Health of Argentina. |

*Give information separately for cases and controls in case-control studies and, if applicable, for exposed and unexposed groups in cohort and cross-sectional studies.

**Note:** An Explanation and Elaboration article discusses each checklist item and gives methodological background and published examples of transparent reporting. The STROBE checklist is best used in conjunction with this article (freely available on the Web sites of PLoS Medicine at http://www.plosmedicine.org/, Annals of Internal Medicine at http://www.annals.org/, and Epidemiology at http://www.epidem.com/). Information on the STROBE Initiative is available at www.strobe-statement.org.
